# BcSRC2 interacts with BcAPX4 to increase ascorbic acid content for responding ABA signaling and drought stress in pak choi

**DOI:** 10.1093/hr/uhae165

**Published:** 2024-06-21

**Authors:** Zhanghong Yu, Xiaoshan Chen, Zhongwen Chen, Haibin Wang, Sayyed Hamad Ahmad Shah, Aimei Bai, Tongkun Liu, Dong Xiao, Xilin Hou, Ying Li

**Affiliations:** National Key Laboratory of Crop Genetics and Germplasm Enhancement, Key Laboratory of Crop Physiology Ecology and Production Management, Ministry of Agriculture and Rural Affairs, Jiangsu Collaborative Innovation Center for Modern Crop Production, Nanjing Agricultural University, Nanjing 210095, China; National Key Laboratory of Crop Genetics and Germplasm Enhancement, Key Laboratory of Crop Physiology Ecology and Production Management, Ministry of Agriculture and Rural Affairs, Jiangsu Collaborative Innovation Center for Modern Crop Production, Nanjing Agricultural University, Nanjing 210095, China; National Key Laboratory of Crop Genetics and Germplasm Enhancement, Key Laboratory of Crop Physiology Ecology and Production Management, Ministry of Agriculture and Rural Affairs, Jiangsu Collaborative Innovation Center for Modern Crop Production, Nanjing Agricultural University, Nanjing 210095, China; National Key Laboratory of Crop Genetics and Germplasm Enhancement, Key Laboratory of Crop Physiology Ecology and Production Management, Ministry of Agriculture and Rural Affairs, Jiangsu Collaborative Innovation Center for Modern Crop Production, Nanjing Agricultural University, Nanjing 210095, China; National Key Laboratory of Crop Genetics and Germplasm Enhancement, Key Laboratory of Crop Physiology Ecology and Production Management, Ministry of Agriculture and Rural Affairs, Jiangsu Collaborative Innovation Center for Modern Crop Production, Nanjing Agricultural University, Nanjing 210095, China; National Key Laboratory of Crop Genetics and Germplasm Enhancement, Key Laboratory of Crop Physiology Ecology and Production Management, Ministry of Agriculture and Rural Affairs, Jiangsu Collaborative Innovation Center for Modern Crop Production, Nanjing Agricultural University, Nanjing 210095, China; National Key Laboratory of Crop Genetics and Germplasm Enhancement, Key Laboratory of Crop Physiology Ecology and Production Management, Ministry of Agriculture and Rural Affairs, Jiangsu Collaborative Innovation Center for Modern Crop Production, Nanjing Agricultural University, Nanjing 210095, China; National Key Laboratory of Crop Genetics and Germplasm Enhancement, Key Laboratory of Crop Physiology Ecology and Production Management, Ministry of Agriculture and Rural Affairs, Jiangsu Collaborative Innovation Center for Modern Crop Production, Nanjing Agricultural University, Nanjing 210095, China; National Key Laboratory of Crop Genetics and Germplasm Enhancement, Key Laboratory of Crop Physiology Ecology and Production Management, Ministry of Agriculture and Rural Affairs, Jiangsu Collaborative Innovation Center for Modern Crop Production, Nanjing Agricultural University, Nanjing 210095, China; National Key Laboratory of Crop Genetics and Germplasm Enhancement, Key Laboratory of Crop Physiology Ecology and Production Management, Ministry of Agriculture and Rural Affairs, Jiangsu Collaborative Innovation Center for Modern Crop Production, Nanjing Agricultural University, Nanjing 210095, China

## Abstract

As a reducing substance, ascorbic acid functioned well in abiotic and biotic stress. However, the regulatory mechanism of drought resistance is rarely known in pak choi. Here we found a gene *BcSRC2* containing a C2 domain that responds to ABA signal and drought regulation in pak choi. Silencing of *BcSRC2* reduces ascorbic acid content and drought resistance of pak choi. In *Arabidopsis*, BcSRC2 overexpression promotes ascorbic acid accumulation and increases drought tolerance. Meanwhile, transcriptome analysis between WT and *BcSRC2*-overexpressing pak choi suggests that ascorbic acid-related genes are regulated. BcSRC2 interacts with BcAPX4 and inhibit APX activity *in vitro* and *in vivo*, increasing the ascorbic acid content. We also found that drought stress increases ABA content, which reduces the expression of BcMYB30. BcMYB30 bound to the promoter of *BcSRC2* and reduced its expression. Overall, our results suggest that a regulatory module, BcMYB30-BcSRC2-BcAPX4, plays a central role in increasing ascorbic acid content for responding ABA-mediated drought regulation in pak choi.

## Introduction

Pak choi [*Brassica campestris* (syn. *Brassica rapa*) ssp. *chinensis*] comes from China and is popular around the world. Pak choi can provide a balanced diet rich in fiber, soluble sugars and vitamins [1, 2]. However, salt, drought, extreme temperature, abiotic and biotic stresses influence the quality and productivity of pak choi [3, 4]. Drought stress causes water loss and affects plant growth and quality [5]. When water is scant, plants accumulate higher levels of reactive oxygen species (ROS), further impairing cell development and enzyme activity and leading to plant death [6]. Greater ROS productions cause enzyme inactivation, protein degradation and membrane lipid peroxidation [7].

Drought tolerance involves complex biological processes involving different response pathways. Drought stress accumulates abscisic acid (ABA). ABA affects stomatal closure to keep water in the plants. In plants, ABA transduces drought signals to ABA receptors, like PYRABACTIN RESISTANCE/PYR1-Like /Regulatory Components of ABA Receptor proteins. These ABA receptors interact with clade A protein phosphatases type 2C to form stable subsequences. The interaction complexes activate sucrose nonfermenting 1-related protein kinase 2, which further phosphorylates regulated genes [8–11]. The PYR/PYL/RCAR-PP2Cs-SnRK2s signaling pathway has been confirmed in several studies and is involved in MYB transcription factor genes [12, 13]. In *Arabidopsis*, MYB2 bind to the promoter of *drought-responsive 22* to participate in drought regulation [14]. AtMYB30, AtMYB60 and AtMYB96 act as an ABA signaling cascade to regulate drought stress by participating in redox oxidation [15–17].

ROS contain H_2_O_2_, OH^−^, and O_2_^−^ and are produced by unfavorable growth conditions [18]. Plants have evolved many methods to scavenge ROS, including nonenzymatic antioxidants (ascorbate, glutathione, and vitamin E) and enzymatic antioxidants (APX, GPX, CAT, SOD, and POD) [19]. Ascorbate protects cells from oxidative stress [20]. AsA has been confirmed to react directly with OH^−^ and O_2_^−^ to produce H_2_O and H_2_O_2_ [21]. AsA also acts as a reducing agent to produce e^−^, which is used by APX to eliminate H_2_O_2_ [22, 23]. In *Arabidopsis*, low AsA content increased sensitivity to environmental stresses [24]. The regenerative metabolism of the ascorbic acid cycle has been well studied; how it responds to ABA signaling and drought stress is not yet clear. For pak choi, it is crucial to explore mechanisms of ascorbic acid regulation by ABA and drought signaling.

In this study, a previously identified gene, *BcSRC2*, containing a C2 domain was characterized. Silencing of *BcSRC2* significantly reduced AsA content and increased APX activity compared to control plants during drought treatment in pak choi. Transcriptome analysis between *BcSRC2*-overexpressed plants and wild type plants revealed an ascorbic acid metabolism-related gene, *BcAPX4*. BcSRC2 was found to interact with BcAPX4 and regulated its APX enzyme activity. These results showed that *BcSRC2* upregulated ascorbic acid content by inhibiting APX activity to combat drought. Here, we also found that *BcSRC2* responded to the ABA signal and reduced the sensitivity to ABA. In addition, a negative ABA response gene, BcMYB30, could bind to the promoter of *BcSRC2* and reduce its expression. Based on the BcMYB30-BcSRC2-BcAPX4 regulatory module, our study proposes a possible regulatory mechanism of ABA and drought resistance through AsA metabolism in pak choi.

## Results

### Characterizations of *BcSRC2*

**Figure 1:**
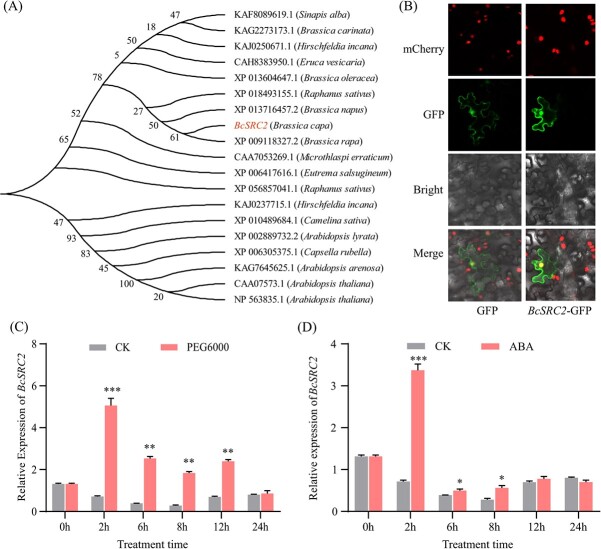
Characterization pattern of *BcSRC2*. **A** Phylogenetic tree analysis of *BcSRC2*, its homologs and orthologs from various plants. Bootstrap values were replicated 1000 times, and substitution distance was 0.02. **B** Subcellular localization of BcSRC2 in tobacco leaves. Bar = 20 μm. **C**–**D** Expression of *BcSRC2* at 0, 2, 6, 8, 12, and 24 h under PEG6000 treatment (**C**) or ABA treatment (**D**). *BcGAPC* was used as an internal reference. Student’s *t*-test was used to detect the significant difference (^*^P < 0.05, ^**^P < 0.01, ^***^P < 0.001).

Our previous study identified a gene associated with AsA content regulation. Sequence comparison of this gene revealed high homology to *Arabidopsis AtSRC2*, thus it was named *BcSRC2* (Fig. 1A). The results of phylogenetic tree analysis demonstrated that *BcSRC2* was conserved and had high similarity with other species (Fig. 1A). *BcSRC2* encoded a putative protein with a C2 domain, which may be associated with intracellular proteins [25]. Transient expression experiment suggested that BcSRC2 expressed in the nucleus and cytoplasm (Fig. 1B). Under PEG6000 and ABA treatment, *BcSRC2* was expressed higher than control groups (Fig. 1C and D). It implied that *BcSRC2* might response to drought regulation and ABA signaling.

### Silencing *BcSRC2* decreases ascorbate content and drought tolerance

**Figure 2:**
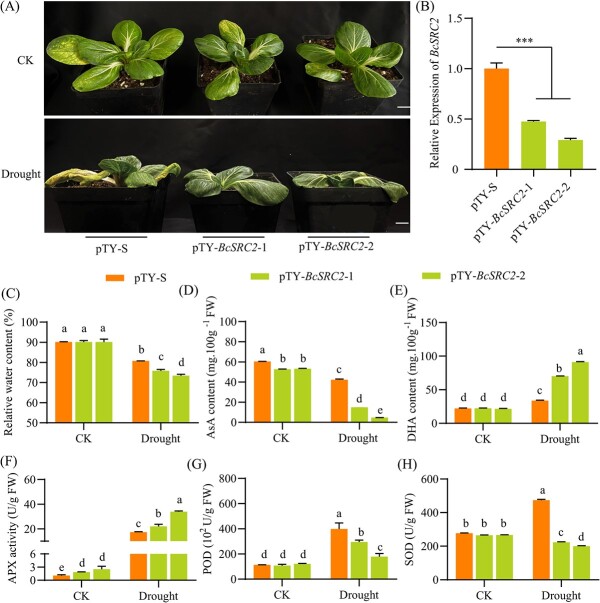
Silencing of *BcSRC2* reduces drought tolerance in pak choi. **A** Phenotype of *BcSRC2*-silenced and control pak choi under natural drought for 15 days. Bar = 2 cm. **B** Silent efficiency identification of *BcSRC2* between pTY-S and pTY-*BcSRC2* pak choi by qPCR. *BcGAPC* worked as an internal reference. Student’s *t*-test for detecting significant difference (^***^P < 0.001). (**C**) Relative water content, (**D**) AsA content, (**E**) DHA content, (**F**) APX activity, (**G**) POD, and (**H**) SOD activity were measured in pTY-S and pTY-*BcSRC2* after drought stress. For **C**–**H**, two-way ANOVA determined the significant differences (*P* < 0.05).

Two silent lines, pTY-*BcSRC2*–1 and pTY-*BcSRC2*–2, were generated using virus-mediated silencing systems for drought treatment (Fig. 2A; Fig. ure S1, see online supplementary material). With significant silencing efficiency, pTY-S and pTY-BcSRC2 plants were treated with natural drought stress (Fig. 2A and B). After drought treatment, the relative water content decreased more in *BcSRC2*-silenced plants than control (Fig. 2C). This suggests that drought caused more damage to BcSRC2-silent strains in pak choi. The silent lines of *BcSRC2* had lower ascorbic acid content than pTY-S under drought stress (Fig. 2D). Meanwhile, the DHA content and APX activity increased more in pTY-*BcSRC2* group than control (Fig. 2E and F). The enzyme activity of POD and SOD was reduced in *BcSRC2*-silencing plants after drought treating (Fig. 2G and H). These results indenticated that silencing of *BcSRC2* generated AsA reduction by increasing APX enzyme activity. It shows that silencing of *BcSRC2* reduces the tolerance to drought in pak choi.

Drought-treated overexpression of *BcSRC2* was then carried out in *Arabidopsis* (Fig. S2A and B, see online supplementary material). Transgenic plants obtained higher chlorophyll content and relative water content (Fig. S2C and D, see online supplementary material), lower MDA content and lower APX activity (Fig. S2E and F, see online supplementary material), higher AsA content and POD activity (Fig. S2G and H, see online supplementary material) than the wild type under drought. These demonstrate that the ectopic expression of *BcSRC2* increased drought tolerance in *Arabidopsis*.

### Overexpressing *BcSRC2* increases ascorbic acid content in pak choi

**Figure 3:**
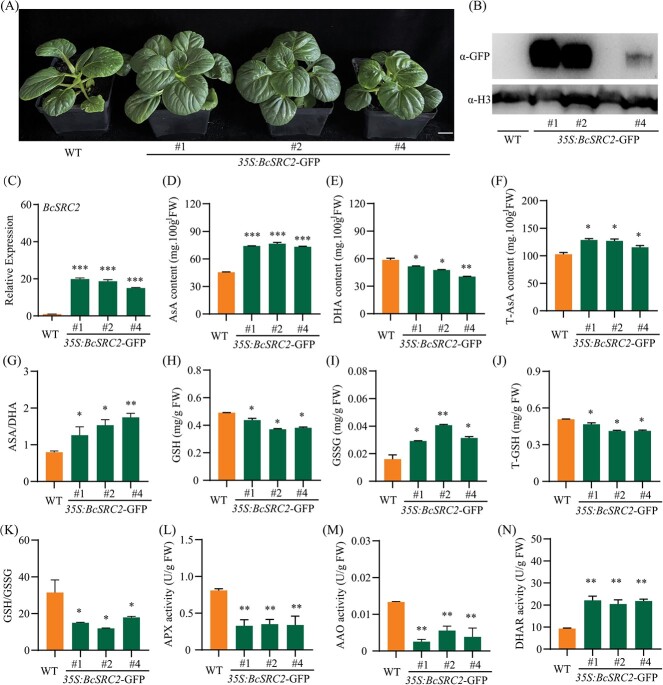
Overexpression of *BcSRC2* increasing AsA content in pak choi. **A** Phenotype of *BcSRC2*-overexpressing pak choi. Bar = 2 cm. **B** Identification of BcSRC2 protein in WT and overexpressing pak choi by western blot. Anti-H3 acted as an internal reference. **C** The expression of *BcSRC2* in WT and transgenic pak choi by qPCR. *BcGAPC* was used as loading control. (**D**) AsA content, (**E**) DHA content, (**F**) total AsA content, (**G**) AsA/DHA, (**H**) GSH content, (**I**) GSSG content, (**J**) total GSH content, (**K**) GSH/GSSG, (**L**) APX activity, (**M**) AAO activity, (**N**) DHAR activity were measured in WT and BcSRC2-OE pak choi. For **C**–**N**, Student’s *t*-test calculated significant difference (^*^P < 0.05, ^**^P < 0.01, ^***^P < 0.001).

The transgenic pak choi were confirmed by western blot and qPCR (Fig. 3A–C, Fig. S7A). Several antioxidants and antioxidant enzyme activities were measured in *BcSRC2-*overexpressing lines (OE) lines and WT. In *BcSRC2* overexpressed pak choi, the content of AsA and total AsA increased, DHA content decreased (Fig. 3D–F). AsA/DHA increased in *BcSRC2*-OE compared to WT (Fig. 3G). The antioxidant content (GSH and total GSH) decreased, whereas the oxidant content (GSSG) increased upon the overexpression of *BcSRC2* pak choi (Fig. 3H–J). Meanwhile, the ratio of GSH/GSSG decreased in BcSRC2-OE lines (Fig. 3K). This suggests that BcSRC2 overexpression may have an impact on the AsA-GSH pathway in pak choi. Furthermore, APX and AAO enzyme activities were decreased, while DHAR enzyme activity was induced in *BcSRC2*-OE lines compared with WT (Fig. 3L–N). It proves that *BcSRC2* participates in the accumulation of pak choi AsA.

### BcSRC2 inhibits APX activity by interacting with BcAPX4

**Figure 4:**
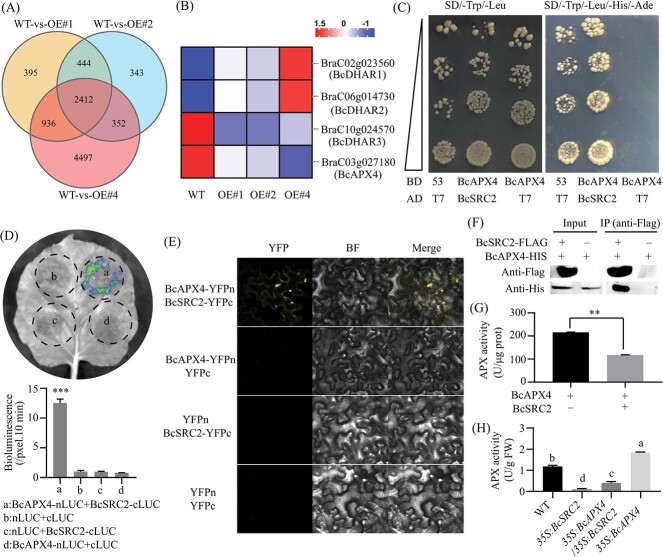
BcSRC2 interacts with BcAPX4 to inhibit APX activity. **A** Venn analysis between WT and *BcSRC2*-overexpressing pak choi. **B** Heatmap analysis of ascorbic acid-related genes from transcriptome analysis. **C** The yeast-two-hybrid confirms that BcSRC2 interacts with BcAPX4. **D** For the luciferase complementation imaging (LCI) assay, different areas of tobacco leaves were co-injected using different combinations. Only the group of BcSRC2-cLUC and BcAPX4-nLUC showed bright fluorescence. Student’s *t*-test determined significant differences (^***^P < 0.001). **E** For bimolecular fluorescent complementation (BiFC) assay, BcSRC2 interacted with BcAPX4. YFP: yellow fluorescent protein; BF: bright field; Merge: overlay of YFP and BF. **F** Co-immunoprecipitation (Co-IP) assay was used for the interaction between BcSRC2 and BcAPX4. Protein from group (BcSRC2-Flag and BcAPX4-His) and group (BcAPX4-His) was extracted, and immunoprecipitated with anti-Flag antibody. Anti-Flag detected BcSRC2-Flag and anti-His detected BcAPX4-His. **G** APX enzyme activity was measured in the group with BcAPX4 protein and the group with BcAPX4 and BcSRC2 proteins, respectively. **H** APX activity is measured between different combinations through transient expression in pak choi leaves. For **G**–**H**, one-way ANOVA determined significant differences determined by *P* < 0.05.

Differences in gene expression between wild type and overexpressing plants revealed that 2412 genes were regulated (Fig. 4A). Overall, we identified 1425 upregulated and 987 downregulated genes in overexpressing plants compared to control plants (Fig. S3A, see online supplementary material). There were three genes related to the AsA-GSH pathway (*BcDHAR1*, *BcDHAR2*, *BcAPX4*) with the same trend as the DHAR and APX activity profiles, which were selected for further investigation (Fig. 4B).

We hypothesized that there might be a factor related to BcSRC2. Therefore, a Y2H assay was performed. It confirmed that BcSRC2 could interact with BcAPX4 (Fig. 4C). The LCI assay showed clear luciferase signals in the BcSRC2-BcAPX4 group compared with the other groups (Fig. 4D). A BiFC assay confirmed that BcSRC2 could interact with BcAPX4 (Fig. 4E). Co-immunoprecipitation assay identified that BcAPX4-His could be immunoprecipitated with BcSRC2-Flag (Fig. 4F, Fig. S7B). It suggests that BcSRC2 could interact with BcAPX4 in plants.

Furthermore, exploring the effect of BcSRC2-BcAPX4 interaction on APX enzyme activity, BcSRC2 and BcAPX4 proteins were purified using prokaryotic expression. APX enzyme activity was higher in the BcAPX4 protein groups than in the BcSRC2, BcAPX4 protein group (Fig. 4G). The H_2_O_2_ content served as a substrate reference and showed no obvious change with different treatments (Fig. S3B, see online supplementary material). Furthermore, the effect of BcSRC2-BcAPX4 interaction on APX enzyme activity in plants was verified by the transient expression method (Fig. S3C, see online supplementary material). APX activity increased in 3*5S: BcAPX4* plants while decreasing in *35S: BcAPX4*/*35S: BcSRC2* plants compared with WT (Fig. 4H). Meanwhile, APX activity was lowest in *35S: BcSRC2* plants (Fig. 4H). It suggests that BcSRC2 could interact with BcAPX4, resulting in inhibition of APX enzyme activity.

### Silencing *BcAPX4* enhances drought tolerance

**Figure 5:**
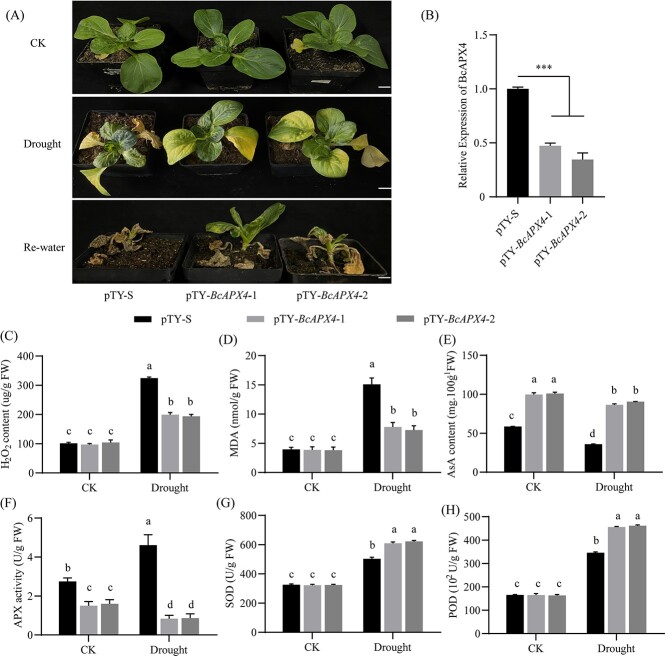
Silencing of *BcAPX4* enhances drought tolerance in pak choi. **A** Phenotype of pTY-S and pTY-*BcAPX4*-silence pak choi in response to natural drought for 15 days and rewatering for 15 days. Bar = 2 cm. **B** Silencing efficiency detection of *BcAPX4* in pTY-S and pTY-*BcSRC2* pak choi by qPCR. *BcGAPC* acted as an internal reference. Student’s *t*-test calculated the significant difference (^***^P < 0.001). Content of (**C**) H_2_O_2_, (**D**) MDA, and (**E**) AsA, activity of (**F**) APX, (**G**) SOD, and (**H**) POD were measured in pTY-S and *BcAPX4* silent pak choi after drought stress. For **C**–**H**, significant differences were determined by two-way ANOVA (*P* < 0.05).

After measuring silencing efficiency, pTY-S and pTY-*BcAPX4* plants were treated with natural drought for 15 days (Fig. 5A and B). After drought treatment, the leaves of pTY-S plants dried out while the leaves of pTY-*BcAPX4* plants began to turn yellow (Fig. 5A). After 10 days of rewatering, the *BcAPX4*-silenced plants showed green leaves, whereas the control plants did not turn green (Fig. 5A). Under drought stress, the H_2_O_2_ and MDA contents of *BcAPX4*-silenced plants were lower than that of control (Fig. 5C and D, Table S2). AsA antioxidant content was increased in *BcAPX4* silent plants compared with control after drought stress (Fig. 5E). The APX, SOD, and POD activities showed different trends under drought stress. Drought stress treatment reduced APX activity in *BcAPX4*-silenced lines compared with that in control (Fig. 5F). Compared with pTY-S, the SOD and POD activities were increased in pTY-*BcAPX4* (Fig. 5G and H). All results suggest that silencing *BcAPX4* alleviated drought stress damage by affecting antioxidants, and antioxidant enzymes in pak choi.

### Silencing *BcSRC2* reduces sensitivity to ABA

**Figure 6:**
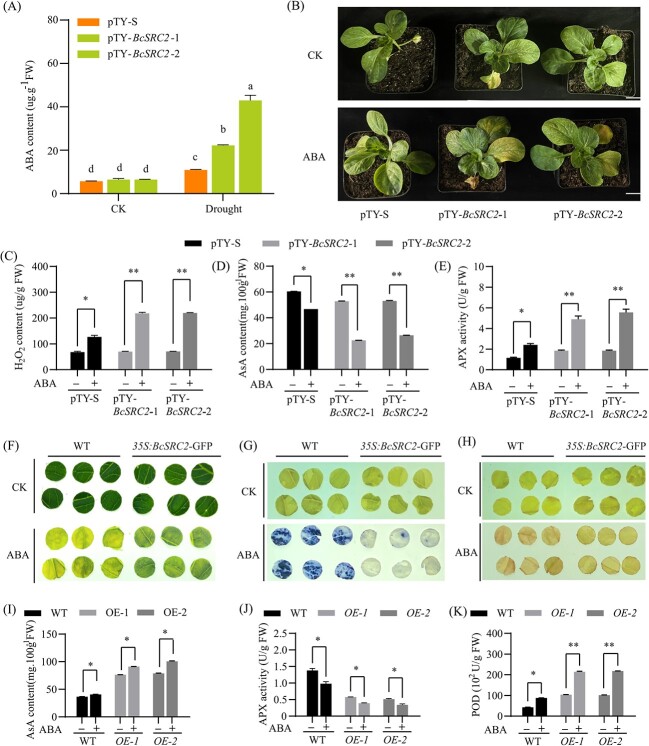
Performance of control, *BcSRC2* silencing, and *BcSRC2*-OE under ABA treatment in pak choi. **A** The ABA content measured in pTY-S and pTY-*BcSRC2* pak choi. Letters above the bars indicated significant differences which were determined by two-way ANOVA (*P* < 0.05). **B** Phenotypes between pTY-S and pTY-*BcSRC2* treated with 100 μM ABA for 5 days. Bar = 2 cm. **C**–**E** H_2_O_2_, AsA content, and APX activity of control and *BcSRC2*-silenced pak choi before and after ABA treatment. **F** Phenotypes of WT and *BcSRC2-OE* pak choi under 100 μM ABA for 5 days. **G**–**K** NBT, DAB coloring, AsA content, APX, and POD activity of WT and *BcSRC2-*overexpressed pak choi before and after ABA treatment. For (**C**–**E**, **G**–**K**), Student’s *t*-test measured significant difference (^***^P < 0.001).

According to PLANTPAN3.9 analysis, the promoter of *BcSRC2* (2000 bp from the ATG start codon) had ABRE *cis-*elements related to ABA signaling (Fig. S4, see online supplementary material). In our study, ABA treatment increased BcSRC2 expression (Fig. 1D). Drought stress could increase ABA content (Fig. S5A, see online supplementary material). The increase in the ABA content in *BcSRC2* silent lines was greater than that in control (Fig. 6A). This suggested that *BcSRC2* could respond to the ABA signal. For further investigation, control plants and *BcSRC2*-silenced plants were treated with ABA. Under ABA treatment, the leaves of pTY-*BcSRC2* turned yellow, whereas those of pTY-S were green (Fig. 6B). ABA treatment caused a higer H_2_O_2_ content in pTY-*BcSRC2* than pTY-S (Fig. 6C). AsA content was decreased both pTY-*BcSRC2* and pTY-S after ABA treatment. The decrease of AsA content in *BcSRC2* silent plants was greater than that in control (Fig. 6D). ABA treatment increased APX activity in pTY-*BcSRC2* and pTY-S. The increase in APX activity was higher in *BcSRC2* silent cells than in control cells (Fig. 6E). These results indicated that silencing BcSRC2 enhances ABA sensitivity in pak choi.

Furthermore, *BcSRC2*-OE plants showed greener leaves than control plants after ABA treatment (Fig. 6F). *BcSRC2*-OE plants processed lower levels of ROS than WT (Fig. 6G and H). This implied that *BcSRC2* transgenic plants suffered less damage. Meanwhile, *BcSRC2*-OE exhibited much higher AsA content, lower APX activity, and higher POD activity under ABA treatment (Fig. 6I–K). These showed that the overexpressing *BcSRC2* reduced the sensitivity of pak choi to ABA.

Then WT, *BcSRC2*-OE *Arabidopsis*, and *src2* mutants were treated with or without ABA (Fig. S5B, see online supplementary material). After treatment with ABA, *src2* mutants germinated and produced small roots without green cotyledons. The wild type germinated and produced little roots and cotyledons. Meanwhile, *BcSRC2*-OE plants displayed more primary roots, lateral roots, and aerial parts (Fig. S5C, see online supplementary material). Greening rates of the WT, *BcSRC2*-OE lines, and *src2* under ABA treatment were measured. On MS medium, the greening rates of WT, *BcSRC2*-OE *Arabidopsis,* and *src2* lines were similar (Fig. S4D and E, see online supplementary material). After treatment with ABA, the greening rate was higher in *BcSRC2*-overexpressed lines than in WT and lowest in *src2* mutants (Fig. S5D and E, see online supplementary material).

**Figure 7:**
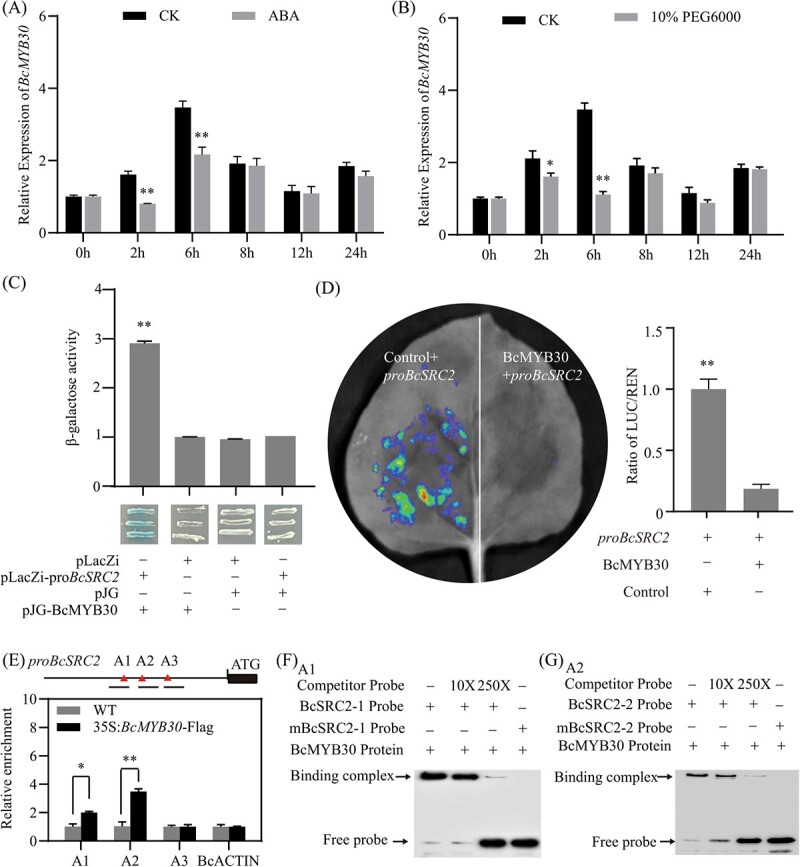
BcMYB30 directly binds to the promoter of *BcSRC2* and inhibits its expression. **A**, **B** Expression of BcMYB30 under ABA treatment (**A**) and PEG6000 treatment (**B**) in pak choi. **C** Y1H and β-galactosidase activity assay confirm that BcMYB30 binds to the promoter of *BcSRC2*. **D** LUC assay indicates that BcMYB30 could reduce the *BcSRC2* expression. Control: 35S. **E** ChIP-qPCR assay showing the relative numbers of *BcSRC2* fragments in *35S*: *BcbHLH30*-FLAG seedlings. **F**, **G** For EMSA assays, labeled with/without biotin and mutant probes were co-incubated with BcMYB30 protein. In the mutant probe, the putative box was replaced with AAAAAA. 10X and 250X represent the rates of the competitor. For **A**–**E**, a significant difference was calculated by Student’s *t*-test (^*^P < 0.05, ^**^P < 0.01).

The WT, *BcSRC2*-OE, and *src2* lines grew on soil and were treated with an ABA solution (Fig. S5F, see online supplementary material). Under ABA treatment, the increase of MDA content in *BcSRC2*-OE lines was less than that in WT, which was highest in *src2* mutants (Fig. S5G, see online supplementary material). AsA content increased in *BcSRC2*-OE lines and WT, whereas it decreased in *src2* mutants (Fig. S5H, see online supplementary material). Notably, the *BcSRC2-*OE lines showed a greater increase in AsA content than the WT (Fig. S5H, see online supplementary material). Under ABA treatment, APX enzyme activity decreased in the *BcSRC2*-OE and WT lines, whereas it increased in *the src2* mutants (Fig. S5I, see online supplementary material). The decrease of APX activity was greater in *BcSRC2*-overexpressed plants than in WT (Fig. S5I, see online supplementary material). It was suggested that *BcSRC2* reduced sensitivity to ABA, possibly by increasing AsA content to eliminate the oxidation damage.

### BcMYB30 directly binds to the *BcSRC2* promoter and inhibits *BcSRC2* expression

Studies have shown a strong connection between ABA-mediated drought stress and MYB family transcription factors [16]. *MYB30* has been reported to be an ABA-negative responder, whose expression level was decreased under ABA [17]. Here, the expression of *BcMYB30* was reduced under ABA and PEG6000 treatment in pak choi (Fig. 7A and B). This implied that *BcMYB30* was involved in ABA-mediated drought stress in pak choi. To explore whether *BcMYB30* affects *BcSRC2*, a Y1H assay was designed to describe that BcMYB30 binds to the promoter of *BcSRC2* (Fig. 7C).

To further verify how BcMYB30 regulates the expression of *BcSRC2*. Dural-LUC reporter assays were used. The BcSRC2_pro_::LUC and 35S_pro_::BcMYB30 group produced lower luminescence signals than the control group (Fig. 7D). It indicated that BcMYB30 suppressed *BcSRC2* gene expression. There were three *cis*-element regions in the *BcSRC2* promoter, designed A1 (831–835 bp), A2 (860–865 bp), A3 (1071–1076 bp) (Fig. 7E). The chip-qPCR assay revealed that BcMYB30 bound to the promoter of *BcSRC2* at two ABRE sites, A1 (831–835 bp) and A2 (860–865 bp) (Fig. 7E; Fig. S6, see online supplementary material， Fig. S7C). A band shift was observed when BcMYB30-His protein was incubated with biotin primers in EMSA assay (Fig. 7F and G). The DNA-protein complex gradually faded with the decrease of biotin primers. No mark shift was detected when the *cis*-element ACGTG was mutated to AAAAA. It demonstrates that BcMYB30 directly binds to the *BcSRC2* promoter and inhibits the expression of *BcSRC2*.

## Discussion

Drought is a worldwide environmental problem. Natural drought decreases food production and harms human health. Drought stress reduces the water content and produces more ROS to damage cells and plants [26]. Increased ROS causes membrane lipid peroxidation, which disrupts the redox balance in plants [27]. Antioxidant and antioxidant enzyme systems are activated in plants during dehydration. Ascorbic acid, as a weak acid, can produce H^+^ in cells, which directly react with superoxide anions and mitigate the toxicity of oxygenated metabolites derived from superoxide anions [28, 29]. In tomatoes, exogenous AsA solution increased the AsA pool to protect roots from drought damage [30]. Seeds treated with AsA can decrease ROS damage induced by drought in cauliflower [31]. As reported, ascorbic acid also acted as a coenzyme factor and participated in enzymatic reactions to eliminate oxides and generate water [29, 32]. Exogenous AsA enhanced the SOD and POD activity to alleviate the drought damage in maize [33]. In our study, *BcSRC2* reduced drought stress injury by increasing AsA content, SOD activity, and POD activities in pak choi and *Arabidopsis* (Fig. 2; Fig. S2, see online supplementary material). This suggests that multiple pathways work synergistically to defend against environmental stress in plants.

In pak choi, BcSRC2 overexpression affected the antioxidant and antioxidant enzyme activities in the AsA-GSH pathway (Fig. 3). Using transcriptome analysis, *BcAPX4* was strongly correlated with *BcSRC2* (Fig. 4A and B). Furthermore, our results identified that BcSRC2 interacted with BcAPX4 and inhibited the APX activity (Fig. 4C–H). These results demonstrated that *BcSRC2* increased AsA content by inhibiting APX enzyme activity, which further mitigated the damage of drought. Meanwhile, silencing of *BcAPX4* could enhance drought tolerance in pak choi (Fig. 5). This is similar to the finding that knockout *AtAPX1* decreased APX activity and increased the AsA content to enhance resistance to Selenium stress in *Arabidopsis* [34]. These demonstrated that BcAPX4 acts as the core target gene of BcSRC2 involved in drought regulation. However, other studies have also shown that enhancing APX activity was beneficial in improving plant stress resistance. In *Eleusine coracana*, drought stress significantly induced APX and MDHA activity which is used to scavenge H_2_O_2_ and MDA [35]. In sweet potato, higher SOD and APX activity enhanced drought tolerance [36]. It might be related to the severity of drought stress. Under moderate drought (less than 8 d), the enzyme activity of APX increased; when suffering from severe drought (more than 8 d), the enzyme activity of APX decreased significantly [37]. Previous studies have proposed that APX eliminating ROS occurs at the beginning and middle of a drought. During severe drought, plant chloroplast and APX functions were impaired. At this stage, the enzymatic systems are represented by SOD and POD, which play the main role in eliminating ROS [38, 39]. These showed that the regulation mechanism of drought resistance in plants was complex and diverse.

Previous studies have demonstrated that plants respond to drought stress through ABA-dependent and ABA-independent pathways [40–43]. It is consistent with our results that drought stress could produce more ABA content in pak choi (Fig. S5A, see online supplementary material). The ABA content was higher in the *BcSRC2*-silenced plants than in control plants (Fig. 6A), suggesting that the silencing of *BcSRC2* resulted in increased ABA sensitivity in the plant. In pak choi, ABA caused more yellow leaves in *BcSRC2* silent plants than in control (Fig. 6B). The higher H_2_O_2_ content, APX activity, and lower AsA content indicated that *BcSRC2*-silenced plants were sensitive to ABA (Fig. 6C–E). After ABA treatment, *BcSRC2*-overexpressed plants accumulated less ROS content in pak choi (Fig. 6F–H). The increase in AsA content, POD activity, and decrease in APX activity in *BcSRC2-*OE lines suggested that BcSRC2 overexpression reduced ABA sensitivity in pak choi. Meanwhile, the increase of MDA content was highest in the *src2* mutant, followed by the WT, and lowest in *BcSRC2*-OE plants (Fig. S5G, see online supplementary material). This suggested that *BcSRC2*-OE lines experienced less oxidative damage caused by ABA. Here, ABA treatment decreased APX activity in WT and *BcSRC2*-OE lines and increased it in *src2* mutants (Fig. 5H). AsA content increased in WT and *BcSRC2*-OE lines but decreased in *src2* mutants (Fig. 5I). It also found that ABA increases AsA content under drought stress in maize [44].

MYB family factors participate in abiotic stress responses to ABA signals [45, 46]. In *Arabidopsis*, *AtMYB44* resisted drought damage by regulating ABA-mediated stomatal closure [58]. Degradation of *AtMYB30* is induced by RHA2b for responding ABA signaling [17]. MYB73 interacts with PYL3 and is then induced by the ABA content [47, 48]. These indicated that MYB family genes were closely related to ABA-mediated drought stress. In pak choi, the expression of *BcMYB30* decreased under ABA and drought stress (Fig. 7A and B). It implied that *BcMYB30* might respond to ABA-mediated drought regulation. In this study, the Y1H, LUC, CHIP-qPCR, and EMSA assays confirmed that *BcMYB30* directly bound to the ABRE-binding site of the *BcSRC2* promoter and decreased its expression. It demonstrated that ABA-mediated drought regulation decreased the expression of *BcMYB30*, which further released *BcSRC2*.

**Figure 8:**
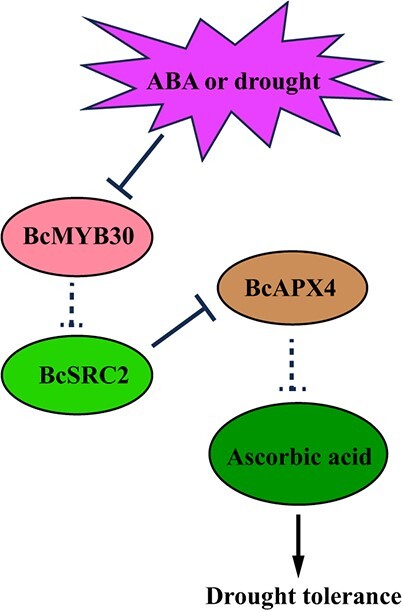
Proposed model of *BcSRC2* enhances tolerance to ABA-mediated drought stress by increasing AsA content in pak choi. In the presence of drought and ABA, the expression of *BcMYB30* could be increased. The inhibitory effect of BcMYB30 on *BcSRC2* was attenuated. This resulted in increased expression of *BcSRC2*. The increased expression of *BcSRC2* enhanced the inhibition of APX by interacting with *BcAPX4*. It could further improve the ascorbic acid content to enhance the drought tolerance.

In conclusion, an AsA-positive regulator *BcSRC2* was isolated and cloned into pak choi. Overexpression of *BcSRC2-*induced AsA content while silencing of *BcSRC2* reduced AsA content, which, through interaction with BcAPX4 inhibited its activity. Furthermore, the ABA-negative regulator BcMYB30 bound to the promoter of *BcSRC2* and decreased its expression. Finally, we proposed a potential regulatory mechanism, BcMYB30-BcSRC2-BcAPX4, for responding ABA signaling and drought stress in pak choi (Fig. 8).

## Materials and methods

### Plant material and treatments

Pak choi cultivar ‘Suzhouqing’ was used for the following assays. Pak choi, *Nicotiana benthamiana*, and *Arabidopsis thaliana* (Col-0) were grown in plant chambers (16 h light at 24°C and 8 h dark at 18°C).

For PEG6000 treatment or ABA treatment, 10% PEG6000 solution or 100 μM ABA solution were used to spray the two-week-old pak choi. Water was used as a control. Leaves were harvested at 0, 2, 6, 8, 12, and 24 h. Then, samples were stored at −80°C for further study.

To study the response of *BcSRC2* and *BcAPX4* to drought stress in pak choi, *BcSRC2*-silenced, *BcAPX4*-silenced plants and pTY-S plants were used and subjected to natural drought treatment. BcSRC2-overexpressed Arabidopsis and WT were treated with drought stress.

To analyse the effect of ABA on *BcSRC2*, control plants, *BcSRC2*-silenced and *BcSRC2*-overexpressed pak choi were treated with 100 μM ABA solution. Meanwhile, WT, *BcSRC2*-overexpressed and *src2* Arabidopsis were treated with 10 μM ABA solution.

### Genetic transformation of pak choi and *A. thaliana*

The open reading fame of *BcSRC2* was fused into pEarlyGate103 plasmid to obtain overexpression vector (Primers listed in Table S1, see online supplementary material). This combined vector was transformed into *Agrobacterium tumefaciens*. Transgenic Arabidopsis were produced using the floral dip method [49]. Transgenic plants were confirmed through qPCR assay.

*Agrobacterium* contained 35S: *BcSRC2*-GFP vector was also used to transform into pak choi. The detail protocol was described as in a previous report [50]. In brief, pak choi seeds were sterilized and planted in 1/2 MS medium. After geminating, seedlings were cut off and kept in pre-culture medium. The explants were infected by *Agrobacterium* and placed in co-medium at dark. Then injected explants were transferred into differentiation medium. Further, these explants were selected under screening medium and rooting medium for 30 days, respectively. Finally, transgenic plants were identified by western blot assay. Transgenic pak choi and WT were grown in a greenhouse for further analysis.

### Silencing of *BcSRC2* and *BcAPX4* in pak choi

A 40 bp fragment of *BcSRC2* and *BcAPX4* was designed and reversed complementarity to form an 80 bp palindrome structure using GenScript (Nanjing, China) (sequences are listed in Table S1, see online supplementary material). pTY-S vector and pTY-BcPDS vector were used as negative control and positive control, respectively. Two-weeks seedlings were used for mosaic virus-mediated gene silencing assay [51, 52]. The plants suffering from gene gun were placed under 22°C/18°C, 16 h–8 h light/dark. After one month, plants with mosaic symptoms had RNA extracted to analyse the silencing efficiency following SYBR® Green Premix Pro Taq HS qPCR Kit II (AG, Changsha, China). After confirming, silent plants and control plants were used for further assay.

### Yeast two-hybrid assay

The ORF of BcSRC2 and BcAPX4 was cloned into pGBKT7 and pGADT7 vector, respectively (primers are listed in Table S1, see online supplementary material). The recombinant constructs, pGADT7-BcSRC2 and pGBKT7-BcAPX4, were co-transformed into Y2H Gold cells, and then grown on SD/−Trp/−Leu media, SD/−Trp/−Leu/-His/−Ade media for 3 days. Single-positive clones were diluted 10 times (10×), 100 times (100×), and 1000 times (1000×). pGBKT7-Lam and pGADT7-T were used as negative controls. Positive controls were pGADT7-T and pGBKT7–53.

### Bimolecular fluorescence complementation assays

For bimolecular fluorescence complementation assays, the ORF of the BcAPX4 was fused with N-terminal YFP, whereas the ORF of BcSRC2 was fused with C-terminal YFP (primers are listed in Table S1, see online supplementary material). The YFPc-*BcSRC2* and YFPn-*BcAPX4* were transformed into *Agrobacterium* and then co-infected in 4-week tobacco leaves. The YFP fluorescence signals were observed using a Zeiss LSM780 confocal microscope after 3 days (Zeiss LSM 780).

### Luciferase complementation imaging assays

According to the previous protocol, luciferase complementation imaging (LCI) was performed to confirm the BcSRC2-BcAPX4 interaction. The recombined vector, nLUC-BcAPX4 and cLUC-BcSRC2, were constructed and transformed into *Agrobacterium*, respectively (primers are listed in Table S1, see online supplementary material). The other groups, nLUC+cLUC-BcSRC2, nLUC-BcAPX4 + cLUC, nLUC+cLUC, acted as negative controls. Different combinations of *Agrobacterium* were co-expressed in 4-week-old leaves. After 6 hours, tobacco leaves were sprayed with 100 mM D-luciferin and kept in the dark for 5 min (Yeasen, Shanghai, China). Then a charge-coupled device camera was used to observe the fluorescent signal.

### Co-immunoprecipitation assays

The Co-IP assay protocol was described in the previous study [53]. Here, *35S:BcSRC2-*FLAG, *35S:BcAPX4-*HIS were constructed and transformed into *Agrobacterium* for infecting tobacco leaves (primers are listed in Table S1, see online supplementary material). After expressing for 60 hours, the protein was extracted and then immunoprecipitated with anti-FLAG M2 magnetic beads (Sigma-Aldrich, St Louis, MO, USA). After washing, the samples were analyzed with anti-Flag (Sigma-Aldrich) and anti-His (Abcam, America) antibodies.

### Yeast one-hybrid assay and β-galactosidase activity

For yeast one-hybrid (Y1H) assay, the ORF of *BcMYB30* were cloned into pJG vector. The promoter of *BcSRC2* was fused into pLacZi vector (primers are listed in Table S1, see online supplementary material). The pIG-BcMYB30 and pLacZi-proBcSRC2 were co-transformed into EGY48 strain. The transformed cells were cultured on the SD-Trp/-Ura medium for 3 d at 28°C and then placed on SD/−Trp/-Ura/Gal/Raf/X-Gal (20 μg/mL) medium for 3–5 d. The combinations, pLacZi and pIG-BcMYB30, pLacZi and pJG, pLacZi-proBcSRC2 and pJG, were negative controls. The β-galactosidase Assay Kit (Beyotime, Beijing, China) was used to measure the β-galactosidase activity in different groups.

### Transient dual-luciferase assays

For luciferase (LUC)/Renilla (REN) assays, the promoter of *BcSRC2* was inserted into pGreenII 0800-LUC vector as reporter, the ORF of BcMYB30 was constructed into overexpression vector as effectors (primers are listed in Table S1, see online supplementary material). Then the recombined vectors were transformed into *Agrobacterium* and co-expressed in 4-week-old tobacco leaves. The luciferase signal was observed using a charge-coupled device (CCD) camera after spraying with 100 mM D-luciferin. The LUC and REN activity were measured following the Dural-luciferase Reporter Assay System (Yeasen, Shanghai, China).

### Electrophoretic mobility shift assay

The ORF sequence of BcMYB30 was fused into pET-28a vector and then transformed into *BL21* strain to extract BcMYB30-His proteins using HisSeq Ni-NTA Agarose gel (Beyotime, Beijing, China). Two 24-bp probes of *BcSRC2* promoter labelled with or without biotin label were designed and synthesized from the company (Sangon, Shanghai, China). The core *cis*-element, ACGTG, was turned to AAAAA as a mutant probe. The fusion protein and probes were incubated at 24°C for 30 min and then separated using a chemiluminescent EMSA kit (Thermo Fisher, California, USA).

### Chromatin immunoprecipitation assay

The ChIP assay was described following the previous method [54]. In brief, 2 g *BcMYB30*-overexpressed pak choi leaves were treated with 0.1% formaldehyde for cross-linking the protein-DNA complexes. After isolation and ectraction, the supernatants were immunoincubated with Flag-antibody (Sigma-Aldrich). Then the protein-DNA complex was reversed and further DNA purification according to the EpiQuikTM Plant ChIP Kit (Base Catalog # P-2014-24, Epigentek, New York, USA). The purified DNA was used as templates for qPCR. Primers are listed in Table S1 (see online supplementary material).

### Determination of ascorbic acid, abscisic acid content, and AsA-GSH pathway enzyme activities

The ascorbic acid content, DHA content, and total AsA content were extracted and measured using the HPLC system following the previous protocol [55]. POD and SOD activity were measured using the kit (Sangon, Shanghai, China).

After drought treatment, the leaves of control and silent lines were collected and extracted with 80% methanol overnight. Abscisic acid content was measured as described previously using a Liquid Dhromatography Coupled to Triple Quadrupole Mass Spectrometryto (Triple Quad 6500+, AB Siex) [56].

The enzyme activities in AsA-GSH pathway, GSH, GSSG, AAO, and DHAR were measure following the kits’ protocols (Solarbio, Beijing, China). The ascorbate peroxidase activity (APX) consumed ascorbic acid to remove hydrogen peroxide using SpectraMax Paradigm. According to the decreasing value of OD_290_ per min, the APX activity was calculated. The solution was mixed with 0.1 mM EDTA-Na_2_, 5 mM AsA, 20 mM H_2_O_2_, and 40 μL samples and then immediately determined the change of OD_290_ value in 1 min at 20°C.

### DAB, NBT staining and malondialdehyde (MDA), H_2_O_2_ content and relative water contents (RWCs) measurements

The rosette leaves of WT and overexpression plants after 3 days ABA treatment were used for DAB and NBT staining. Briefly, leaves were cut into bottles and soaked in DAB buffer (1 mg/ml) or NBT buffer (0.5 mg/ml) in the dark at 100 rpm for 12 h. Then leaves were transferred into 95% ethanol and boiled at 95°C for 10 min. Finally, the color changes of leaves were observed. The MDA content and H_2_O_2_ content were measured as per the kits (Solarbio, Beijing, China). RWC was calculated following the previous study [57].

## Supplementary Material

Web_Material_uhae165
