# Supplementary material for: BcSRC2 interacts with BcAPX4 to increase ascorbic acid content for responding ABA signaling and drought stress in pak choi
Source: Hortic Res. 2024 Jun 21;11(8):uhae165. doi: 10.1093/hr/uhae165 (PMC11784589; doi:10.1093/hr/uhae165)
Supplement: Web_Material_uhae165 [file web_material_uhae165.zip › minor revision-Supplementary Figure.docx]

**Supplementary Figure**

**
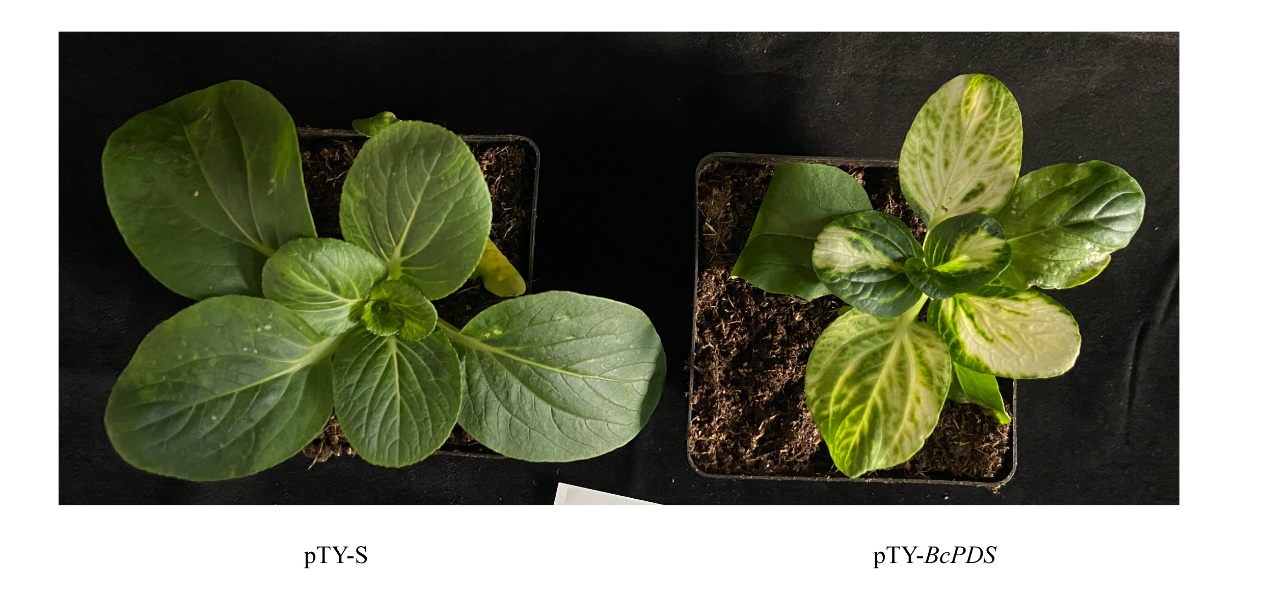
**

**Supplementary Fig. S1 TYMV-mediated silencing system in Pak-choi**

Phenotypes of pTY-S and pTY-*BcPDS* plants; bar = 2 cm.


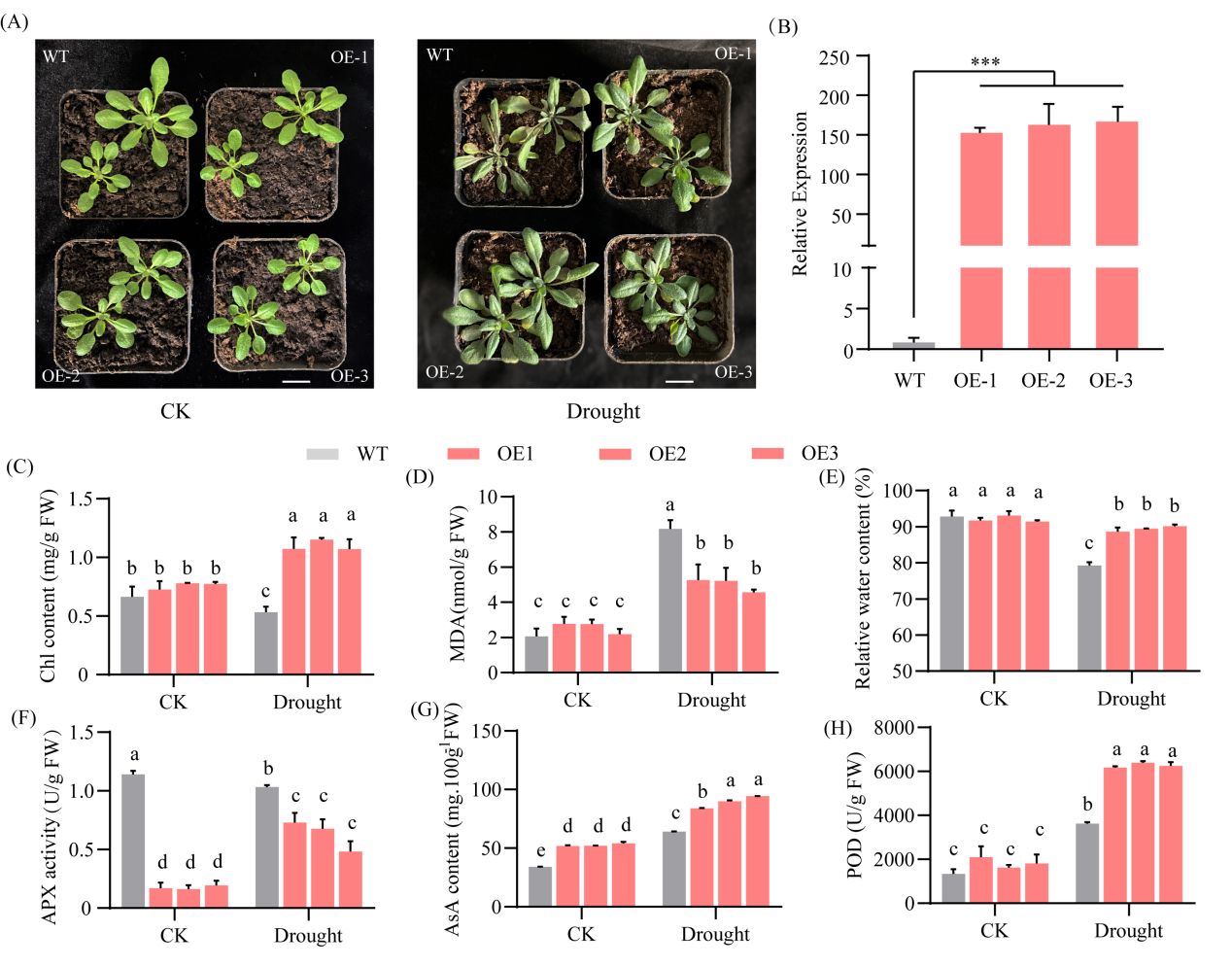


**Supplementary Fig. S2 Overexpression of *BcSRC2* enhances drought tolerance in *Arabidopsis***

(A) Phenotype of *BcSRC2-*overexpressing *Arabidopsis* in response to drought stress. The control plants and *BcSRC2*-overexpressing lines grow for three weeks and are treated with natural drought stress for 9 days. Bar=2 cm. (B) Expression of *BcSRC2* was detected in transgenic *Arabidopsis*. *BcGAPC* was used as the internal reference. The significant difference was determined by the Student’s t-test (^***^*P* <0.001). (C) Chl content, (D) MDA, (E) Relative water content, (F) APX enzyme, (G) AsA content, and (H) POD were measured in WT and *BcSRC2*-OE *Arabidopsis* lines before and after drought treatment. For (C)-(H), letters above the bars indicate significant differences, which were determined by two-way ANOVA (*P*< 0.05).


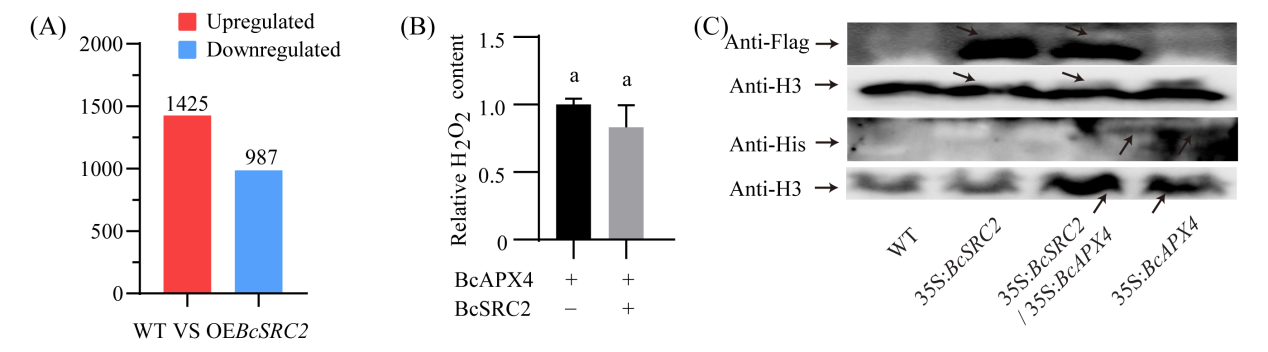


**Supplementary Fig. S3** (A) Number of upregulated or downregulated genes in the comparison between WT and *BcSRC2-*OE transgenic plants. (B) H_2_O_2_ content, *in vitro* enzyme activity assay in groups, BcAPX4 protein, BcAPX4, and BcSRC2 proteins. (C) Protein accumulation of BcSRC2-Flag, BcAPX4-His in plants of WT, *35S:BcSRC2*, *35S:BcAPX4*/*35S:BcSRC2*, and *35S:BcAPX4*.


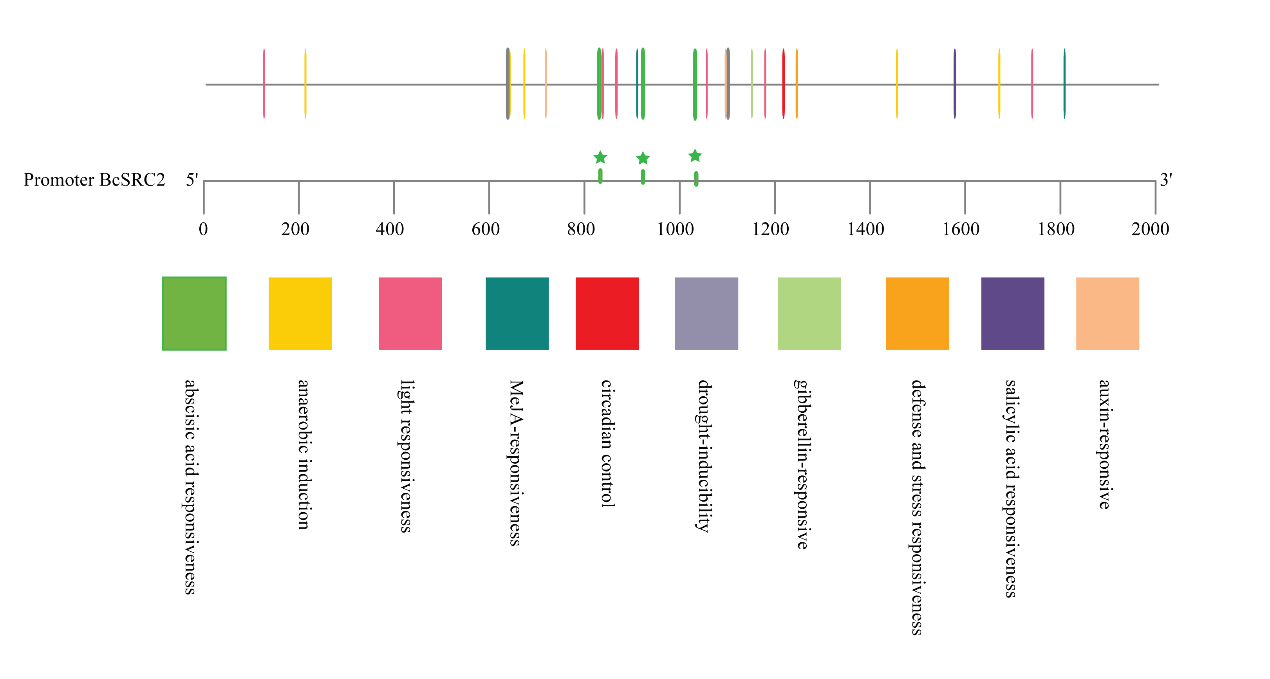


**Supplementary Fig. S4 Prediction of SRC2 promoter binding progenitors**


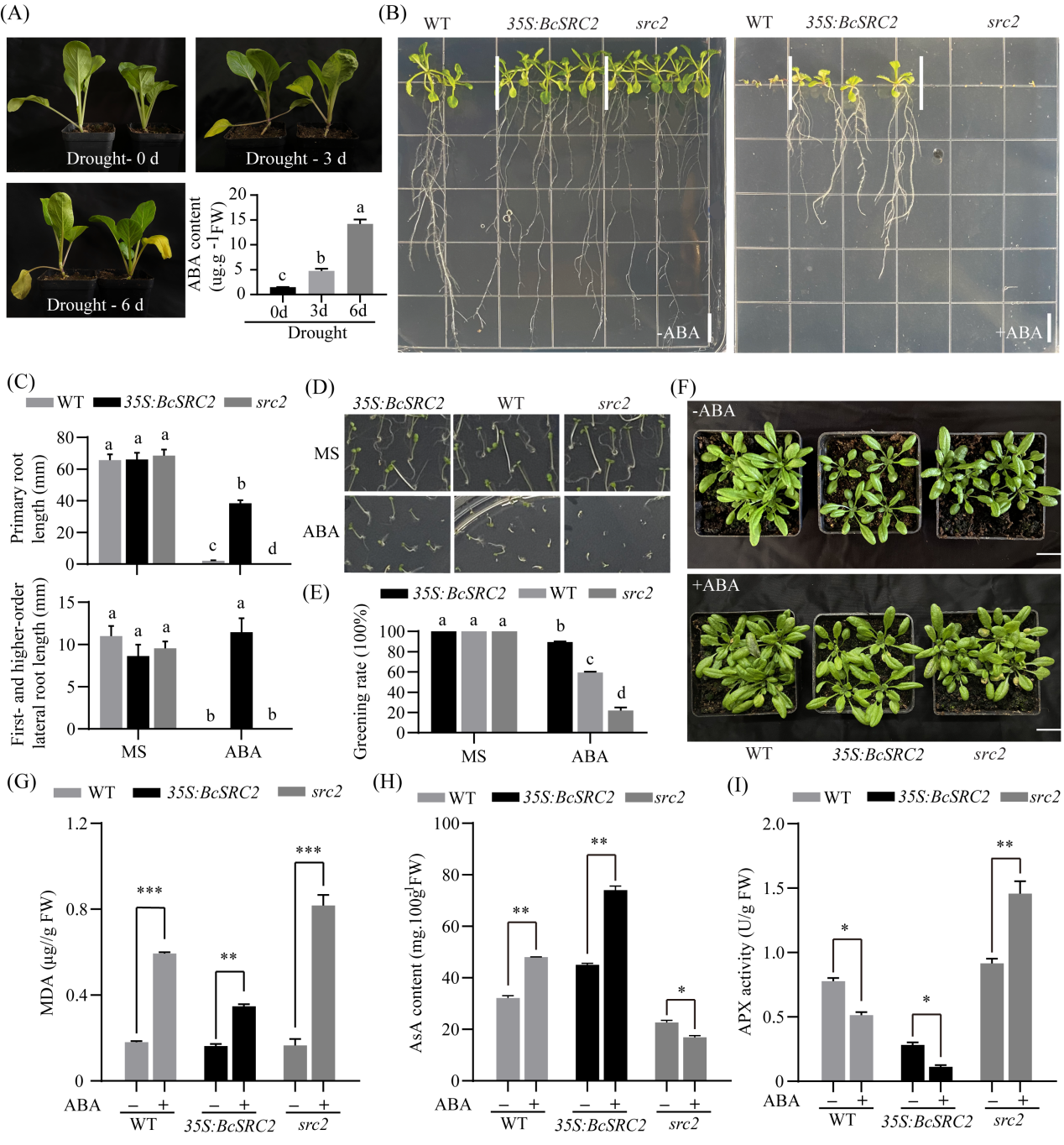


**Supplementary Fig. S5 *BcSRC2* reduced sensitivity to ABA in *Arabidopsis***

(A) ABA content under drought stress in pak-choi. Letters above the bars indicate significant differences determined by one-way ANOVA (*P*< 0.05). (B) Phenotype of WT, *BcSRC2*-OE lines, and *src2* mutants in MS medium with or without ABA. Bar =2 cm. (C) Primary root length and first- and higher-order lateral root length in WT, *BcSRC2*-OE *Arabidopsis,* and *src2* mutants grown on MS medium with or without ABA. (D, E) The seedling phenotype (D) and percentages of greening rate (E) of wild type, *BcSRC2*-OE lines and *src2* plants grown on MS medium with or without ABA. For (A), (C), and (E), the letters above the bars indicate significant differences determined by two-way ANOVA (*P*< 0.05). (F-I) ABA-tolerant phenotypes (F), MDA content (G), AsA content (H), and APX activity (I) of soil-grown WT, *BcSRC2*-OE, and *src2* plants. Three-week-old plants were treated with ABA for 3 days. For (F), Bar = 2 cm. For (G-I), the significant difference was determined by Student’s t-test (^*^*P* < 0.05, ^**^*P* < 0.01, ^***^*P* < 0.001).


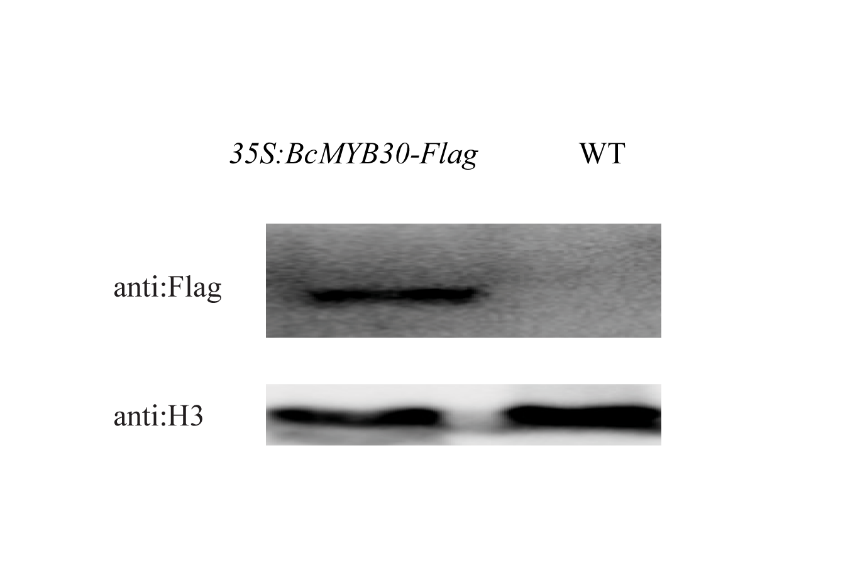


**Supplementary Fig. S6 BcMYB30 protein accumulation in *BcMYB30*-OE lines**

**
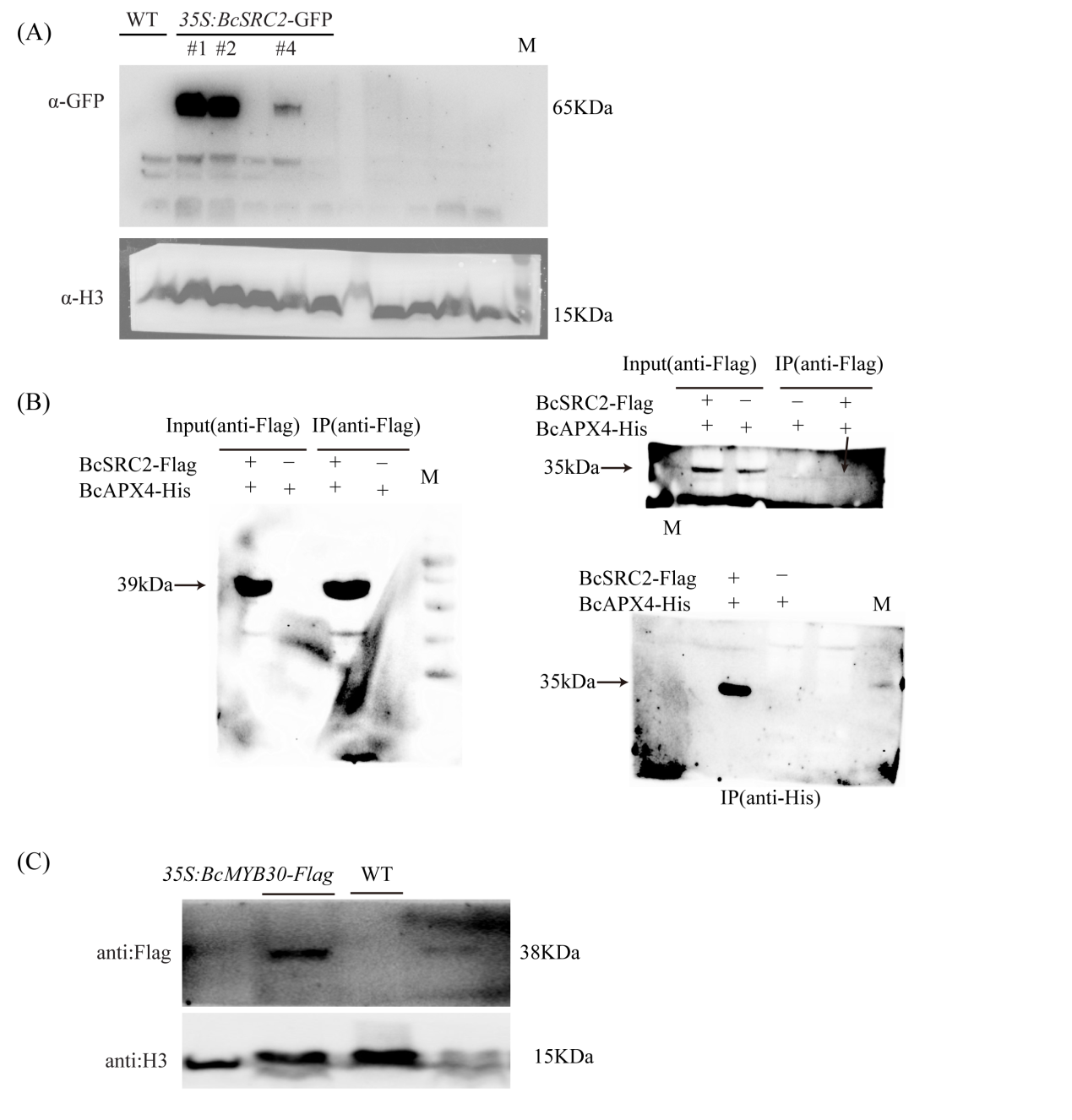
**

**Supplementary Fig. S7 The full dimension for western blot images**

(A) Protein accumulation of BcSRC2 in overexpressing pak-choi and WT. (B) Co-IP assay between BcSRC2 and BcAPX4. (C) Overexpression of BcMYB30 in pak-choi used for Chip-qPCR assay.
